# Specific and safe targeting of glioblastoma using switchable and logic-gated RevCAR T cells

**DOI:** 10.3389/fimmu.2023.1166169

**Published:** 2023-04-14

**Authors:** Haidy A. Saleh, Nicola Mitwasi, Martin Ullrich, Manja Kubeil, Magali Toussaint, Winnie Deuther-Conrad, Christin Neuber, Claudia Arndt, Liliana R. Loureiro, Alexandra Kegler, Karla Elizabeth González Soto, Birgit Belter, Claudia Rössig, Jens Pietzsch, Marcus Frenz, Michael Bachmann, Anja Feldmann

**Affiliations:** ^1^ Helmholtz-Zentrum Dresden-Rossendorf, Institute of Radiopharmaceutical Cancer Research, Dresden, Germany; ^2^ Faculty of Medicine Carl Gustav Carus, Mildred Scheel Early Career Center, Technische Universität Dresden, Dresden, Germany; ^3^ Department of Pediatric Hematology and Oncology, University Children’s Hospital Münster, Münster, Germany; ^4^ Faculty of Chemistry and Food Chemistry, School of Science, Technische Universität Dresden, Dresden, Germany; ^5^ Faculty Informatik and Wirtschaftsinformatik, Provadis School of International Management and Technology AG, Frankfurt, Germany; ^6^ National Center for Tumor Diseases Dresden (NCT/UCC), German Cancer Research Center (DKFZ), Faculty of Medicine and University Hospital Carl Gustav Carus, Technische Universität Dresden, Helmholtz-Zentrum Dresden-Rossendorf (HZDR), Dresden, Germany; ^7^ German Cancer Consortium (DKTK), Partner Site, Dresden, Germany

**Keywords:** CAR T cells, glioblastoma, combinatorial gated targeting, adaptor CAR platform, immunotherapy

## Abstract

Glioblastoma (GBM) is still an incurable tumor that is associated with high recurrence rate and poor survival despite the current treatment regimes. With the urgent need for novel therapeutic strategies, immunotherapies, especially chimeric antigen receptor (CAR)-expressing T cells, represent a promising approach for specific and effective targeting of GBM. However, CAR T cells can be associated with serious side effects. To overcome such limitation, we applied our switchable RevCAR system to target both the epidermal growth factor receptor (EGFR) and the disialoganglioside GD2, which are expressed in GBM. The RevCAR system is a modular platform that enables controllability, improves safety, specificity and flexibility. Briefly, it consists of RevCAR T cells having a peptide epitope as extracellular domain, and a bispecific target module (RevTM). The RevTM acts as a switch key that recognizes the RevCAR epitope and the tumor-associated antigen, and thereby activating the RevCAR T cells to kill the tumor cells. However, in the absence of the RevTM, the RevCAR T cells are switched off. In this study, we show that the novel EGFR/GD2-specific RevTMs can selectively activate RevCAR T cells to kill GBM cells. Moreover, we show that gated targeting of GBM is possible with our Dual-RevCAR T cells, which have their internal activation and co-stimulatory domains separated into two receptors. Therefore, a full activation of Dual-RevCAR T cells can only be achieved when both receptors recognize EGFR and GD2 simultaneously *via* RevTMs, leading to a significant killing of GBM cells both *in vitro* and *in vivo*.

## Introduction

Glioblastoma (GBM) is considered a highly aggressive tumor of the central nervous system (CNS) and the most common type of gliomas, generally associated with poor prognosis and survival (1–3). Despite the advances in research of GBM therapeutics, the standard treatment is still mainly limited to surgical resection of the tumor, radiotherapy and chemotherapy (4–6). Not only, but also the elevated recurrence rate remains as a major drawback of current therapeutic modalities (3, 6, 7). Altogether, these challenges emphasize the urge to a more specific, targeted and less intrusive therapeutic approach.

Immunotherapy represents a valuable option for the treatment of GBM. This includes, but not limited to, antibody (Ab) therapies, cancer vaccines, chimeric antigen receptor (CAR)-expressing immune cells, immune check point inhibitors, myeloid-targeted therapies and others (2, 8–12). To date, none of these has gained an approval for GBM treatment except for a monoclonal Ab, Bevacizumab, which targets vascular endothelial growth factor (VEGF) (13). Nonetheless, several limitations hinder such immunotherapeutic strategies from being clinically beneficial, including the immunosuppressive tumor microenvironment, the heterogeneity of GBM and the therapeutic safety concerns (8, 14, 15). For example, serious side effects were reported after treatment with CAR T cells targeting CD19 or other antigens due to nervous system disorders, cytokine release syndrome or other CAR T cell-associated side effects, such as on-target/off-tumor toxicity against healthy tissues expressing the targeted tumor-associated antigens (TAAs) (16–18). In order to overcome such incidences, novel approaches for safer and more specific therapy are required.

Switchable modular CAR technology is one of the highly promising approaches that showed encouraging results not only in pre-clinical but also in recent clinical studies (19–22). In this study, we present our switchable Reverse CAR (RevCAR) T cell platform. In contrast to conventional CAR platforms, the RevCAR T cell has a peptide epitope in its extracellular domain instead of the Ab-based antigen binding domain. The peptide epitope itself is unable to recognize tumor cells unless a second molecule known as the reverse target module (RevTM) is present (22–24). This RevTM is a bispecific molecule that recognizes the peptide epitope of RevCAR T cells on the one hand and the TAA on tumor cells on the other hand, thereby linking both cells together, which leads to the activation of the RevCAR T cells and, finally to the killing of tumor cells. Thus, the RevTM acts as a switch key that enables the control of RevCAR T cell function which can be switched off upon the elimination of these RevTMs from the body *via* the renal system or the liver, depending on the size and structure of the molecule (22, 24). Until now, we identified two peptide epitopes fulfilling all the requirements to be used in adaptor CAR platforms (22, 24). Both peptide sequences (termed E5B9 and E7B6) are cryptic epitopes taken from the primary amino acid sequence of the nuclear autoantigen La/SS-B with little if any antigenicity including in autoimmune patients (25, 26).

The RevCAR system provides the possibility of multiple targeting (also known as OR gated targeting) (22, 27) by using RevTMs of different specificities, which could be highly valuable in targeting heterogeneous tumors such as GBM. So far, we have shown that the RevCAR system can successfully target cells from prostate cancer and acute myeloid leukemia (23, 24).

In addition to targeting single TAA *via* RevCAR T cells, we here present the results of our dual-targeting approach (also known as AND gated targeting) (23, 24, 27). Dual-RevCARs express two kinds of RevCAR receptors which differ with respect to their extracellular peptide epitopes. Intracellularly, one of the Dual-RevCAR receptors is carrying the CD3 zeta chain signaling motifs (CD3z SD) and the other carries the CD28 co-stimulatory domain (CD28 CSD). According to the AND gate logic of Boolean algebra, a full T cell activation requires the signaling *via* both RevCARs upon recognition of two TAAs simultaneously (23, 24). For that purpose, we developed several novel RevTMs with different structures and characteristics, specific for two highly expressed TAAs on GBM cells: Disialoganglioside (GD2) and the epidermal growth factor receptor (EGFR) (28–34). Both GD2 and EGFR have also been investigated as potential targets in several studies, showing promising therapeutic effect for GBM (9, 35–38). GD2 was shown to be expressed on neurological malignancies, and to be associated with increased invasion and tumorigenicity of cancer cells (32, 36, 39–42). In addition, EGFR overexpression is a common feature of GBM, promoting invasion and aggressiveness of tumor cells (28–30, 43).

In this study, we have successfully applied the RevCAR T cell system for targeting of GBM cells using novel GD2- and EGFR-specific RevTMs. In addition, we are going to present the first successful application of RevCAR T cells for AND gated targeting to eradicate GBM cells both *in vitro* and *in vivo* in a highly efficient, specific and programmable manner.

## Results

### Design of the RevCAR system

**Figure 1:**
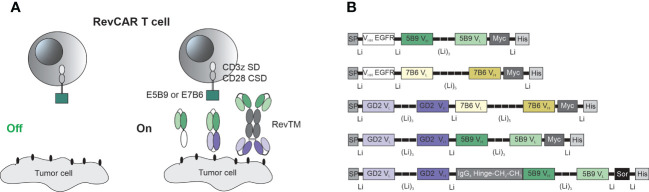
Schematic representation of the RevCAR system. **(A)** RevCAR T cells express reverse chimeric antigen receptor containing an intracellular CD3z signaling domain (SD) and CD28 co-stimulatory domain (CSD), a CD28 transmembrane domain, a hinge and the peptide epitope, either E5B9 or E7B6, as extracellular domains. RevCAR T cells can only be activated upon redirection *via* RevTM to tumor cells. **(B)** Bispecific RevTMs consist mainly of TAA-specific and epitope-specific variable domains that are connected *via* peptide linkers. These binding domains are derived from either a mAb (V_H_, V_L_) or a camelid nanobody (V_HH_). An alternative format of the RevTM contains the hinge and constant domains (CH_2_, CH_3_) of human IgG4 Ab. All molecules contain N-terminal signal peptide (SP) to allow their secretion and C-terminal tags including Myc and 6x Histidine (His) to allow their detection and purification. A sortase cleavage site (Sor) is inserted upstream of the His-tag to enable the removal of the His-tag if required (44).

The RevCAR platform consists of the RevCAR T cell and the RevTM which redirects these cells towards tumor cells and allow the on/off switchability of the RevCAR system (**Figure 1A**). The RevCAR molecules are second-generation CAR constructs, containing the CD3z SD and the CD28 CSD, a transmembrane domain and hinge region derived from the CD28 receptor. Unlike conventional CARs, RevCAR constructs contain a peptide epitope in their extracellular domain instead of an Ab-based binding domain (24). In this study, we have used the two previously described epitopes (E5B9 and E7B6) that are derived from the nuclear protein La-SS/B (25, 45, 46), and therefore named the corresponding RevCARs accordingly: RevCAR-E5B9 or RevCAR-E7B6.

As seen in **Figure 1**, RevTMs are bispecific molecules containing binding domains against the TAA on one side and binding domains against the epitopes E5B9 or E7B6 on the other side. In this study, we have developed five different RevTMs directed against EGFR or GD2. The EGFR-specific RevTMs are based on the camelid variable heavy chain (V_HH_) of a previously used anti-EGFR nanobody (47), linked to the variable domains of the light (V_L_) and heavy (V_H_) chains of either anti-E7B6 or anti-E5B9 monoclonal Abs (mAbs) (**Figure 1B**). In a similar manner, three different GD2-specific RevTMs were developed. These RevTMs contain the V_L_ and V_H_ domains of a previously described conventional anti-GD2 CAR (48), connected to the V_H_ and V_L_ domains specific for the epitopes E5B9 or E7B6. The domains were connected *via* short peptide linkers to provide sufficient flexibility for protein folding.

In addition to the mentioned RevTMs, we have developed an IgG4-based RevTM containing the hinge, CH_2_ and CH_3_ domains of human IgG4 Ab, which were integrated between the anti-GD2 and anti-E5B9 single chain fragment variables (scFvs) (**Figure 1B**). The IgG4-based RevTM is able to form dimers through cysteine residues in the IgG4 backbone (**Figure 1A**).

All RevTMs structure contain a signaling peptide of murine Igκ at the N-terminus to allow the secretion of these molecules. In addition, we have included several tags for detection or purification as schematically shown in **Figure 1B**.

### The binding capability of the novel RevTMs

**Figure 2:**
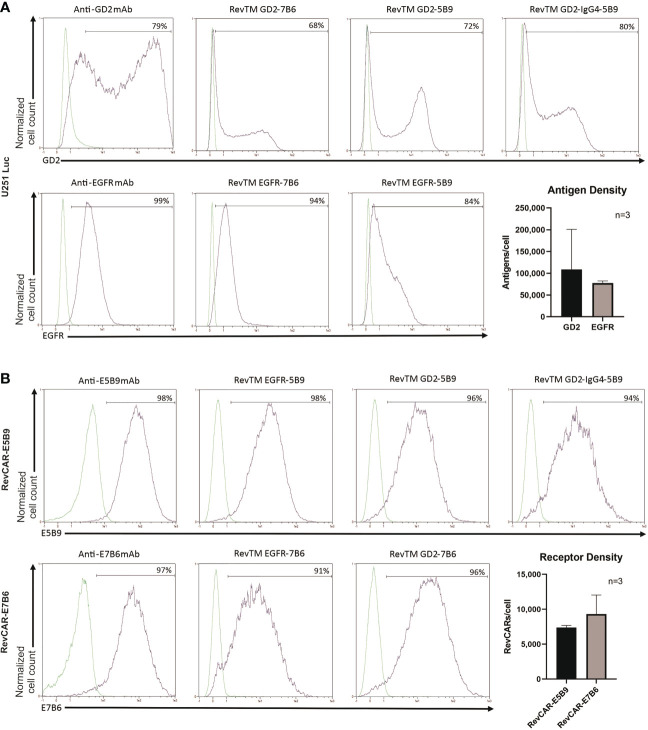
Binding of RevTMs on glioblastoma cells and RevCAR T cells. **(A)** Expression of EGFR and GD2 on U251 Luc cells was confirmed by staining with anti-GD2 and anti-EGFR mAb, and Pacific Blue-goat anti-mouse IgG as a secondary Ab. Moreover, the ability of GD2- and EGFR-specific RevTMs to bind to the surface of U251 Luc cells was detected with APC-conjugated anti-His Ab. The approximate number of GD2 and EGFR molecules present on the cell surface was determined using a bead-based flow cytometry assay. **(B)** Expression of RevCAR-E5B9 and RevCAR-E7B6 was determined on transduced T cells using anti-E5B9 or anti-E7B6 mAb, and Pacific Blue-conjugated goat-anti-mouse IgG as secondary Ab. Binding of the RevTMs to RevCAR T cells was determined using APC-conjugated anti-His Ab. Similarly, number of RevCAR receptors per T cell was determined using bead-based flow cytometry assay. **(A, B)** Quantitative data are shown for three independent experiments as mean ± SD. Flow cytometry data are represented as histograms, where light lines indicate negative or isotype control and dark lines indicate the stained cells.

A successful linkage between RevCAR T cells and tumor cells depends on the functionality of both binding arms of the RevTM. Therefore, we tested the ability of the RevTMs to bind to the targeted TAA on tumor cells and the epitope (E7B6 or E5B9) on RevCAR T cells, respectively. For that purpose, we have used luciferase-expressing glioblastoma cell line (U251 Luc), naturally expressing both EGFR and GD2 on its surface, which was confirmed by staining with anti-GD2 and anti-EGFR mAbs (**Figure 2A**). Moreover, we have estimated the number of both antigens on the surface per cell using a bead-based flow cytometry assay. As shown in **Figure 2A**, U251 Luc cells express higher level of GD2 in comparison to EGFR (approx. 109,000 vs 78,000 antigens per cell, respectively). However, GD2 expression appears to be more variable among GBM cells. All novel EGFR- or GD2-specific RevTMs were able to bind to U251 Luc cells. As observed in **Figure 2B**, all RevTMs were also able to recognize their respective epitope E5B9 or E7B6 on RevCAR T cells, expressing an average of approx. 9,000 E7B6 or 7,000 E5B9 receptors per cell. These binding studies indicate the functionality of both binding domains of the novel RevTMs.

### Specific killing of glioblastoma cells with redirected RevCAR T cells

In both RevCAR systems (E5B9 and E7B6), the ability of the developed RevTMs to specifically redirect RevCAR T cells to lyse tumor cells was evaluated using luminescence-based cytotoxicity assay. For that purpose, we have co-cultured either RevCAR-E5B9 or -E7B6 T cells with U251 Luc cells in the presence or absence of the matching RevTMs, while the non-matching RevTMs were included as a negative control to confirm the specificity of the RevCAR system.

**Figure 3:**
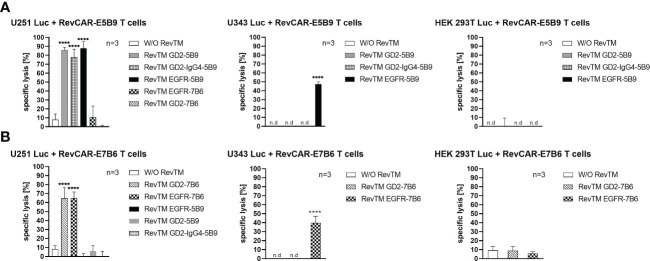
Cytotoxic effect of redirected RevCAR T cells. Luciferase-expressing cells including U251 Luc (GD2^+^/EGFR^+^), U343 Luc (GD2^-^/EGFR^+^) or HEK 293T Luc (GD2^-^/EGFR^low^) were co-cultured with either **(A)** RevCAR-E5B9 or **(B)** RevCAR-E7B6 T cells at effector:target (E:T) cell ratio of 1:4 in the absence or presence of RevTMs (25 nM). After 18-20 h incubation, the cytotoxic activity was measured using luminescence-based cytotoxicity assay. Data are shown for three independent T cell donors and represented as mean ± SD (one-way ANOVA with Dunnett’s multiple comparisons test. (**** p < 0.0001, comparison to samples without RevTM (W/O RevTM), n.d not detected).

As depicted in **Figure 3**, RevCAR-E5B9 T cells were able to induce a significant specific lysis of U251 Luc cells in the presence of either RevTM EGFR-5B9, GD2-5B9 or GD2-IgG4-5B9, where all RevTMs induced a comparable and effective maximum specific lysis. In contrast, only a slight background lysis was observed in the absence of the RevTMs or in the presence of the irrelevant RevTMs EGFR-7B6 and GD2-7B6, indicating the specificity of the RevCAR T cell system. Similarly, RevCAR-E7B6 T cells were only able to induce significant tumor cell killing in the presence of the respective matching RevTMs (EGFR-7B6 and GD2-7B6).

In order to further support their specificity, we have tested both RevCAR systems with other cell lines that lack one or both of our TAAs. For this reason, we have tested the U343 Luc cells which are positive for EGFR but negative for GD2 (**Supplementary Figure 1**) or the HEK 293T Luc cell line which also lacks surface expression of GD2 but has low expression of EGFR (**Supplementary Figure 1**). As shown in **Figure 3**, none of the RevTMs were able to activate RevCAR T cells to lyse HEK 293T Luc cells. As expected, only RevCAR T cells armed with EGFR-specific RevTMs were able to induce lysis in U343 Luc cells.

In conclusion, both RevCAR systems (RevCAR-E5B9 and RevCAR-E7B6) are highly specific, and their activity strictly depends on their cross-linkage with TAA-expressing tumor cells *via* the matching RevTMs, and thus, can efficiently kill GBM cells in a target- and RevTM-dependent manner.

### Estimation of effective working concentrations of the RevTMs

As shown previously, RevCAR T cells are only functional in the presence of the matching RevTM. Therefore, the concentration of the RevTM plays a critical role in the steering and controllability of RevCAR T cells. Here, we have tested a range of the RevTM concentrations in order to determine the effective therapeutic window and to estimate the half-maximal effective concentration (EC_50_).

**Figure 4:**
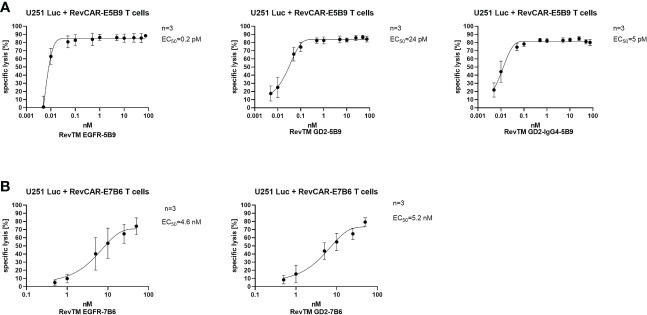
Estimation of the half-maximal effective concentration (EC_50_) of RevTMs. U251 Luc cells were co-cultured with either **(A)** RevCAR-E5B9 or **(B)** RevCAR-E7B6 T cells at E:T ratio of 1:4 in the presence of different concentrations of the indicated RevTMs. After 18-20 h of incubation, tumor cell death was estimated using luminescence-based cytotoxic assay. Data are shown as mean ± SD for three independent T cell donors.

As demonstrated in **Figure 4**, killing *via* the RevCAR T cells occurs in a RevTM concentration-dependent manner. The E5B9-specific RevTMs have EC_50_ values in the picomolar range. Whereas, RevTM EGFR-7B6 and GD2-7B6 have EC_50_ values in the nanomolar range. Moreover, RevTM GD2-IgG4-5B9 has a 5-fold lower EC_50_ in comparison to the smaller scFv-based format of RevTM GD2-5B9.

### Secretion of cytokines by armed RevCAR T cells

**Figure 5:**
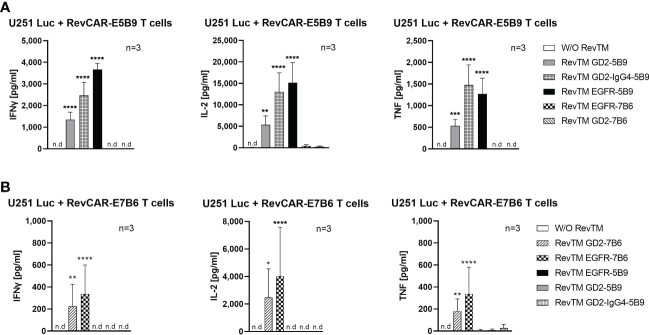
Cytokine secretion from redirected RevCAR T cells. Cytokines including IL-2, IFNγ and TNF were analyzed in the supernatants of co-culture of U251 Luc cells with **(A)** RevCAR-E5B9 or **(B)** RevCAR-E7B6 T cells at E:T ratio of 1:4 in the presence of matching RevTMs or irrelevant RevTMs (negative controls). After 18-20 h, supernatants were harvested and cytokines were detected using ELISA. Results are shown as mean ± SD for three independent T cell donors (* p < 0.0332, ** p < 0.0021, *** p < 0.0002, **** p < 0.0001; comparison to sample W/O RevTM; One-way ANOVA with Dunnett’s multiple comparison test, n.d: not detected).

Upon their activation, T cells secrete several cytokines, which play a vital role in regulating the anti-tumor response as well as the tumor microenvironment. Here, we investigated the secretion of three main pro-inflammatory cytokines, namely IFNγ, TNF and IL-2. As shown in **Figure 5**, redirected RevCAR T cells secrete significantly higher cytokines upon cross-linkage with tumor cells *via* their specific RevTMs compared to the control without RevTM. No or low secretion of cytokines was detected in the presence of the irrelevant RevTMs, indicating the specificity of the system. Interestingly, the EGFR-specific RevTM induces higher cytokines secretion in comparison to the scFv-based GD2-specific RevTMs in both RevCAR platforms. It was also observed that RevCAR-E5B9 T cells secreted more cytokines when armed with RevTM GD2-IgG4-5B9 in comparison to RevTM GD2-5B9.

### Combinatorial targeting of glioblastoma using Dual-RevCAR T cells

**Figure 6:**
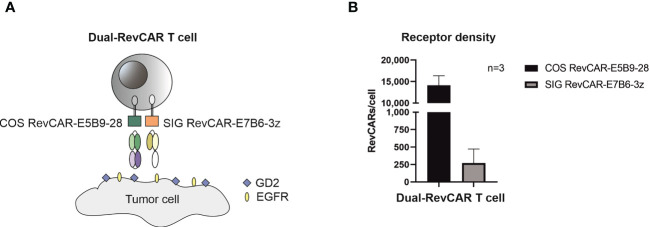
Schematic representation of the Dual-RevCAR system. **(A)** Dual-RevCAR T cells are designed to express two receptors for AND-gate targeting of tumors; one RevCAR having the signaling CD3 zeta domain and E7B6 epitope (SIG RevCAR-E7B6-3z), and the second RevCAR having CD28 co-stimulatory domain and the E5B9 epitope (COS RevCAR-E5B9-28). Dual-RevCAR T cells can be fully activated only when both kinds of RevCARs are cross-linked with tumor cells *via* GD2- and EGFR-specific RevTMs. **(B)** The number of RevCAR receptors on the engineered T cells was estimated using bead-based flow cytometry assay. Results are shown as mean ± SD for three independent T cell donors.

After proving that both RevCAR-E5B9 and -E7B6 systems are highly efficient in targeting GBM, we tested our RevTMs with the Dual-RevCAR T cells in order to achieve a programmable and more specific targeting of GBM. Basically, Dual-RevCARs are designed for AND-gate logic targeting of tumor cells. For that reason, they are modified to express two receptors; one RevCAR receptor contains an intracellular signaling domain of CD3z chain and an extracellular E7B6 epitope (SIG RevCAR-E7B6-3z), while the second receptor contains the co-stimulatory domain of CD28 intracellularly and the E5B9 epitope extracellularly (COS RevCAR-E5B9-28) (**Figure 6A**). Following the combinatorial AND gate logic, a full activation of the Dual-RevCAR T cells would require the signaling *via* both receptors. This could be achieved by simultaneous recognition of GD2 and EGFR with their respective RevTMs. In this study, we have tested the combination of RevTM EGFR-7B6 (activating the SIG RevCAR-E7B6-3z) with either RevTMs GD2-5B9 or GD2-IgG4-5B9 (activating the COS RevCAR-E5B9-28).

After transduction of T cells with bicistronic lentiviral vector encoding both RevCARs, the receptor density was estimated (see Materials and Methods). As shown in **Figure 6B**, more costimulatory receptors (COS RevCAR-E5B9-28, approx. 14,000 receptors/T cell) were expressed on the surface of engineered T cells compared to the number of activating signaling receptors (SIG RevCAR-E7B6-3z, approx. 270 receptors/T cell).

**Figure 7:**
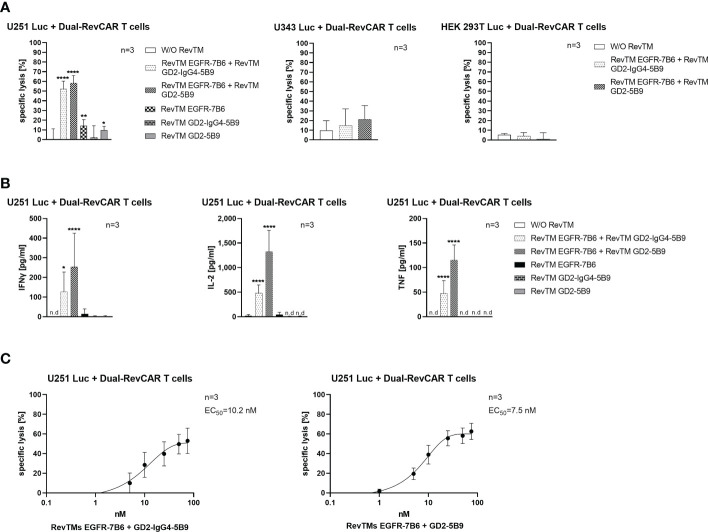
*In vitro* functionality of redirected Dual-RevCAR T cells. **(A)** U251 Luc (GD2^+^/EGFR^+^), U343 Luc (GD2^-^/EGFR^+^) and HEK 293T Luc (GD2^-^/EGFR^low^) were co-cultured with Dual-RevCAR T cells at an E:T ratio of 1:2 in the absence or the presence of 25 nM of each RevTM. **(B)** Supernatants collected from the co-culturing experiment were analyzed for cytokine secretion using ELISA. **(C)** The indicated RevTM combinations were titrated in co-culture with Dual-RevCAR T cells and U251 Luc cells. Results are shown as mean ± SD for three independent T cell donors (* p < 0.0332, ** p < 0.0021, **** p < 0.0001; comparison to sample W/O RevTM; One-way ANOVA with Dunnett’s multiple comparison test, n.d: not detected).

As observed in **Figure 7A**, Dual-RevCAR T cells armed with the combination of RevTM EGFR-7B6 + GD2-5B9 or RevTM EGFR-7B6 + GD2-IgG4-5B9 were able to induce significant tumor lysis. However, application of only one of the RevTMs (EGFR-7B6 or GD2-5B9 or GD2-IgG4-5B9) induced no or negligible background killing. Since we mainly aim to establish a specific and safe immunotherapeutic approach, we further estimated the combinatorial targeting specificity of the Dual-RevCAR T cells by testing their activity on cell lines lacking one or both of the TAAs. As shown in **Figure 7A**, neither U343 Luc nor HEK 293T Luc cell lines were killed when incubated with Dual-RevCAR T cells armed with a single or combination of RevTMs. These data indeed confirm that the tumor cell killing only occurs when both RevCARs are activated. These results are supported by the analysis of secreted cytokines. Dual-RevCAR T cells were able to significantly increase the secretion of IFNγ, IL-2 and TNF, only in the presence of the mentioned RevTMs combinations and U251 Luc cells (**Figure 7B**).

As depicted in **Figure 7C**, the EC_50_ values for the mentioned RevTM combinations were also evaluated, and were found to be effectively functional in the nanomolar range (10.2 nM for RevTM EGFR-7B6 + GD2-IgG4-5B9 and 7.5 nM for RevTM EGFR-7B6 + GD2-5B9).

**Figure 8:**
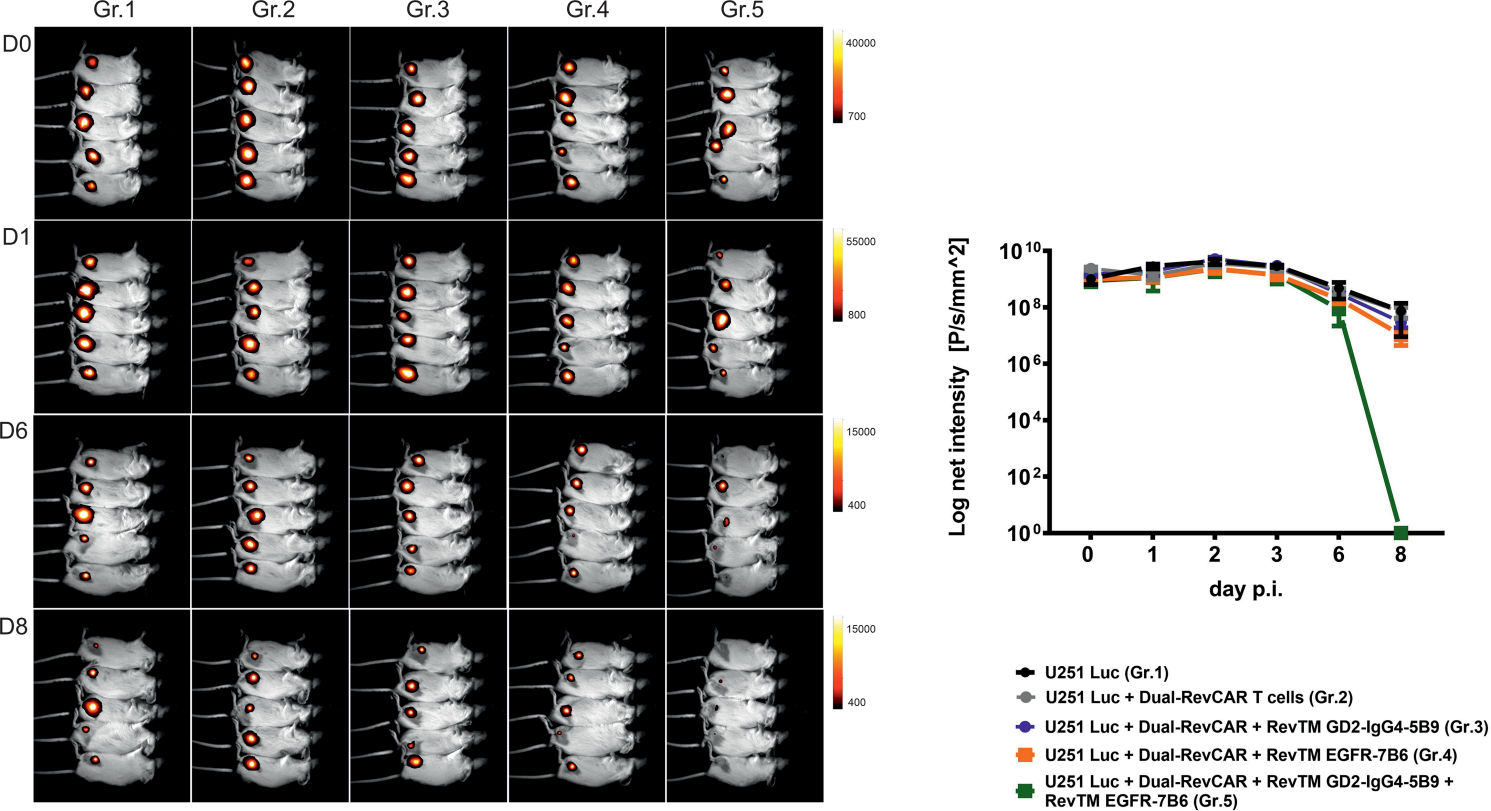
*In vivo* functionality of redirected Dual-RevCAR T cells. Five groups of NXG mice were co-injected with mixtures containing either: (I) U251 Luc alone (Gr.1), (II) U251 Luc and Dual-RevCAR T cells (Gr.2), (III) U251 Luc and Dual-RevCAR T cells in the presence of either RevTM GD2-IgG4-5B9 (Gr.3) or (IV) RevTM EGFR-7B6 (Gr.4) or (V) a combination of both RevTMs (Gr.5). Luminescence was measured over 9 days after intraperitoneal injection of luciferin before each measurement. Day (D0) represents the day of injection of cell mixtures. p.i; post injection of cell mixtures.

In addition to the *in vitro* assays, we also confirmed the dual-targeting effect in a proof-of-concept *in vivo* experiment. Here, five groups of mice were used: The first group was injected with U251 Luc cells alone, the second one was co-injected with tumor cells and Dual-RevCAR T cells, and the third, fourth and fifth groups were co-injected with tumor cells and Dual-RevCAR T cells either with one RevTM or with a combination of two RevTMs (RevTM EGFR-7B6 + GD2-IgG4-5B9) (**Figure 8**). Nine days after the start of the experiment, a clearly reduced luciferase signal was detected in mice treated with the Dual-RevAR T cells and the RevTM combination in comparison to mice lacking RevTM or having received only one RevTM.

### PET imaging of RevTMs in tumor-bearing mice

**Figure 9:**
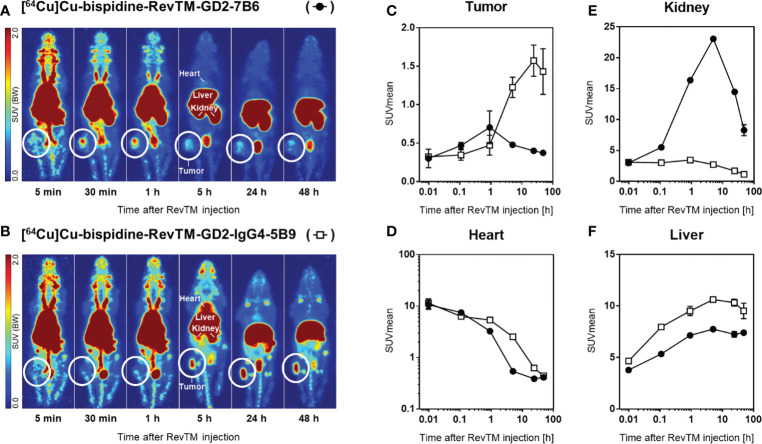
Distribution of radiolabeled RevTMs in U251 Luc tumor-bearing mice. **(A, B)** PET images presented as maximum intensity projections at indicated time points after intravenous injection of [^64^Cu]Cu-bispidine-RevTM-GD2-7B6 or [^64^Cu]Cu-bispidine-RevTM-GD2-IgG4-5B9; white circles indicate the location of the subcutaneous tumor. **(C−F)** Time-activity curves showing the pharmacokinetics of RevTMs in tumor, heart, kidney, and liver. Kinetic data are presented as region-averaged standardized uptake values (SUVmean) with logarithmic time scaling for all analyzed regions and logarithmic SUVmean scaling for the heart.

In order to compare the pharmacokinetic properties of the scFv-based with the IgG4-based RevTMs *in vivo*, both RevTMs were modified with a bispidine chelator and labeled with [^64^Cu]CuCl_2_. Subcutaneous U251 Luc tumors were established in immunodeficient mice. The radiolabeled scFv-based RevTM [^64^Cu]Cu-bispidine-RevTM-GD2-7B6 or the IgG4-based RevTM [^64^Cu]Cu-bispidine-RevTM-GD2-IgG4-5B9 were injected intravenously and visualized *in vivo* using small animal positron emission tomography (PET) imaging (**Figures 9A, B**).

Both ^64^Cu-radiolabeled RevTMs showed uptake in GD2-positive U251 tumors. The tumor uptake was higher for the IgG4-based RevTM than for the scFv-based RevTM (**Figure 9C**). The scFv-based RevTM with a lower molecular weight (~ 57 kDa) showed the maximum uptake in the tumor (SUVmean = 0.7) approximately 40 min after injection followed by continual washout. In contrast, the higher molecular weight IgG4-based RevTM (~ 160 kDa) showed the maximum uptake in the tumor (SUVmean = 1.6) at a later time point, approximately 24 h after injection, which was largely maintained at least until 48 h.

The radiolabeled scFv-based RevTM was eliminated from the blood more rapidly than the IgG4-based RevTM, as determined from time-activity curves in the heart (**Figure 9D**). This observation is mainly attributed to the size differences of the RevTMs and it is in agreement with our previous studies (49–51) and other studies using molecules with similar formats (52). Furthermore, high retention of both RevTMs in the liver contributed considerably to their elimination from the blood.

Both ^64^Cu-labeled RevTMs were excreted *via* both renal and hepatobiliary pathways. Renal excretion of the scFv-based RevTM was associated with a higher kidney retention compared to the IgG4-based RevTM (**Figure 9E**). Hepatobiliary excretion was associated with considerable liver retention for both RevTMs (**Figure 9F**). The IgG4-based RevTM showed the highest liver uptake, possibly due to its Fc portion (53, 54).

## Discussion

Despite the current advances, there is still an urgent need for novel therapeutic strategies for glioblastoma in order to improve survival and combat high recurrence rates (3, 7). Immunotherapies represent a promising approach for targeting GBM, especially CAR T cells which were already approved for clinical application in certain hematological malignancies (55–57). Moreover, promising clinical improvement was observed in glioma patients treated with GD2-specific CAR T cells in a phase 1 clinical study (37). However, special considerations should be taken into account when targeting brain tumors, due to the sensitivity of their location, their heterogeneity and their pathological and molecular characteristics (58–60), which indicates the need for a flexible, specific and safe immunotherapeutic approach.

The concept of modular and switchable CARs has gained a lot of interest in the past few years, and several switchable platforms were developed (19, 22, 61–65). The results obtained from studies with modular CAR platforms indicate that they outperform conventional CARs at least in regards of flexibility and safety. In this study, we presented our switchable RevCAR platform as an approach for targeting GBM with high specificity and improved safety that could aid in the control of on-target/off-tumor or on-target/on-tumor effects by providing a fast safety switch.

The RevCAR system is a modular adaptor CAR T cell platform that can only be activated in the presence of RevTMs. These RevTMs act as bispecific molecules being able to link RevCAR T cells and tumor cells, which leads to T cell activation and tumor killing. In contrast to conventional CAR T cells, RevCAR T cells possess only a short peptide in their extracellular domain (E5B9 or E7B6), which is derived from a human nuclear protein, reducing the risk of immunogenicity (22, 26). This platform also allows multi-targeting of tumors by means of using different RevTMs with different specificities. Moreover, by controlling the half-life and concentration of the RevTM, RevCAR T cell activity can be steered for better safety. This was shown recently in clinical settings (phase 1a trial-NCT04230265) with another modular CAR system, named UniCAR, which was previously developed in our lab (20, 22, 65, 66).

In this current study, we choose to target EGFR and GD2, which were reported to be expressed in GBM and are associated with pathological features of this disease (28, 29, 31, 32). Therefore, we created five different bispecific RevTMs having a nanobody V_HH_ domain against EGFR or an scFv against GD2 on one side, and an scFv against E5B9 or E7B6 on the other side. In addition to the scFv-based formats, we have created a GD2-specific IgG4-based RevTM. Both formats provide a valuable therapeutic tool based on the stage of the disease. Smaller RevTM versions (scFv-based) are eliminated quickly through the renal system as observed in PET imaging, and thereby guaranty a fast off switching of the RevCAR T cells in case of severe side effects that could be anticipated during initial treatment phase. Half-life-extended IgG4-based RevTM could be used in later stages for long-term treatment of residual disease (50, 51). Interestingly, the IgG4-based RevTM showed higher but delayed tumor accumulation in comparison to the scFv-based RevTM, mainly due to the differences in size and half-life in blood. Moreover, PET analysis has shown higher liver accumulation of IgG4-based RevTM, which could be attributed to interaction between the IgG4 Fc with Fc receptors in the liver. This issue could be addressed by using mutated Fc backbone in order to reduce possible binding to Fc receptors (67).

Both, the RevCAR-E5B9 and the RevCAR-E7B6 were able to induce high specific lysis of GBM cells associated with cytokine secretion in a RevTM- and target-dependent manner. However, differences in the efficiency were observed between both RevCARs. The RevCAR-E5B9 was more efficient when combined with its respective RevTMs (EGFR-5B9 or GD2-5B9 or GD2-IgG4-5B9), indicated by EC_50_ values in the picomolar range, whereas the RevCAR-E7B6, armed with RevTMs EGFR-7B6 or GD2-7B6, showed EC_50_ values in the nanomolar range. Moreover, the RevCAR-E5B9 T cells secreted higher amounts of pro-inflammatory cytokines. It is also apparent that the different formats of the RevTM can affect the functionality of RevCAR T cells. As shown in our *in vitro* data, in comparison to the scFv-based RevTM, the IgG4-based RevTM had lower EC_50_ values and induced higher cytokine secretion when combined with its corresponding RevCAR T cells. Such variations could be attributed to differences in the number of binding arms of the molecule as well as the synapse structure between T cells and tumor cells (68–70). Interestingly, we were able to prove that RevCAR T cells can be used in multi-targeting, and when the expression of one antigen is downregulated in tumor cells, other antigens could still be targeted by applying a different RevTM. This hypothesis was affirmed using the U343 Luc GBM cell line which has undetectable GD2 expression but higher EGFR expression. This cell line could be killed only with RevCAR T cells armed with EGFR-specific RevTM. Moreover, cells with only low levels of EGFR and GD2 were not killed by armed RevCAR T cells, indicating the high specificity of the system. It is worth mentioning that EGFR and GD2 are reported to be expressed on many other tumors beside GBM, such as melanoma and neuroblastoma, and therefore, our approach can be extended in the future to target such tumors (32, 71, 72).

Several systems of modular and non-modular gated targeting were described previously demonstrating the difficulties of AND gated targeting (19, 27, 63, 73). However, recently, we have shown feasibility of AND gate tumor targeting using our Dual-RevCAR system which might improve the specificity and safety of CAR T cell therapy (23, 24). Here, we demonstrate an AND gate combinatorial immunotherapeutic targeting against GBM aiming an efficient and safe application in GBM patients. For this purpose, Dual-RevCAR T cells were engineered to express two RevCAR receptors: The first one is the activating signaling receptor with intracellular CD3z signaling domain and extracellular E7B6 peptide, and the second one is the co-stimulatory receptor having the intracellular co-stimulatory domain of CD28 and the peptide E5B9 extracellularly.

As known, a first generation CAR having only CD3z is sufficient to activate T cells to kill tumor cells without co-stimulation. Therefore, our key solution to achieve a true AND gate tumor targeting, where T cells become completely activated only when both the CD3z and CD28 signals are induced upon recognition of two TAAs simultaneously, was the expression level and ratio of the two receptors mediating the activation or the co-stimulatory signal. We have designed both receptors of the Dual-RevCAR system in a way that the activating SIG RevCAR-E7B6-3z is expressed on a very low level whereas the co-stimulatory COS RevCAR-E5B9-28 has much higher expression (24). Based on our data, we are convinced that such low expression of the signaling receptor diminishes the CD3z signal strength in a way that it is not able to induce a full activation of T cells unless a second co-stimulatory signal is provided. As previously shown, due to their smaller size, the two differently structured Dual-RevCAR receptors were encoded bicistronically from a single vector using different signal peptides for their expression into the cell membrane (23, 24). While establishing the Dual-RevCAR system, we have constructed, expressed and tested many different RevCARs varying in their extracellular, hinge, transmembrane and intracellular domains. Furthermore, we have tested different SIG and COS RevCAR pairs encoded either separately or bicistronically. Even though, proteins can be expressed differentially based on their location in the bicistronic vector (74), the expression level of the herein used SIG RevCARs was comparably low when encoded alone on a single vector (24). Based on our data, we found that the optimal expression level and ratio of both SIG and COS RevCARs for a true AND gate targeting does not only depend on the vector design but mainly on the RevCAR structure itself (24).

In this study, we were able to prove that Dual-RevCAR T cells work according to the AND gate principle of Boolean algebra, in which the full activation of RevCAR T cells is achieved only when both receptors get stimulated by the RevTMs directed against EGFR and GD2 at the same time. In the contrary, when one signal is missing, the Dual-RevCAR T cells will show none or minimal activation. In case the SIG RevCAR was triggered alone, the CD3z signal strength was not sufficient to result in a complete T cell activation. The weak CD3z signal had to be fortified with the CD28 co-stimulatory signal derived from the COS RevCAR to induce sufficient signaling leading to the activation of cytotoxic mechanisms and cytokine secretion. Indeed, we were able to show that the combinations EGFR-7B6 and GD2-5B9 or GD2-IgG4-5B9 induce significant elevation in cytokine secretion and *in vitro/vivo* killing of GBM cells in comparison to the groups having single RevTMs. Importantly, when one or both antigens are missing, Dual-RevCAR T cells were not able to induce killing. These results prove that dual-targeting is a highly promising approach to enhance the safety and specificity of the RevCAR system. Interestingly, it appears that combing the scFv-based (GD2-5B9, ~ 57 kDa) and nanobody-based (EGFR-7B6, ~ 45 kDa) RevTM formats is more efficient than combining the larger IgG4-based (GD2-IgG4-5B9, ~160 kDa) RevTM with the nanobody-based format, especially with respect to cytokine release. Such observation seems to be relevant for redirected Dual-RevCAR T cells, where the spatial arrangement of the synapse needs to include two RevCAR receptors and two RevTMs simultaneously, but less critical when using T cells armed with single RevCAR receptor (RevCAR-E5B9 or -E7B6), where the IgG4-based RevTM performed better than the scFv-based format. Importantly, the different RevTM formats can be flexibly combined in order to successfully achieve true AND gate tumor targeting using the Dual-RevCAR approach.

In agreement with other studies (75–80), we have used here a heterotopic mouse model to provide an initial proof-of-concept for the immunotherapeutic functionality of our Dual-RevCAR-based AND gate targeting approach as well as to show an initial proof-of-concept for the accumulation of the RevTMs at the tumor site *in vivo*. As a next step in prospective studies, we aim to confirm the immunotherapeutic and diagnostic imaging potential of the RevCAR system in a more clinically-relevant orthotopic GBM animal model. In principle, the RevTMs can be administrated either intravenously or intracranially together with the RevCAR T cells. An intravenous injection requires that the RevTMs are capable to cross the blood brain barrier (BBB) which might be even a less critical hurdle in heavily pretreated GBM patients that have disrupted BBB (81, 82). However, envisioning clinical application, RevTMs and RevCAR T cells could indeed be intracranially injected in patients as shown previously with other CAR-immune cells (83). The efficacy of such an intracranial approach would be independent from the BBB and the capability of the RevTMs to cross it.

In general, we have shown that the RevCAR system enables targeting of GBM cells effectively with improved controllability, while providing a safety switch and combinatorial targeting approach in order to prevent any therapy-related complications, and to spare healthy tissues that minimally express EGFR and GD2.

## Materials and methods

### Cell lines

HEK 293T, 3T3, and U343 cells were obtained from American Type Culture Collection (ATCC, Manassas, VA, USA). The U251 cell line was provided by Prof. Dr. Dieter Kabelitz (University of Kiel). For further functional assays *in vitro* and *in vivo*, some of these cell lines were genetically transduced with lentivirus to express the firefly Luciferase (Luc). All cells were cultured in DMEM medium, supplemented with 10% fetal bovine serum (FBS), and incubated at 37°C and 5% CO_2_. All cells were confirmed to be free of Mycoplasma.

### Isolation and transduction of human T cells

Human Peripheral Blood Mononuclear Cells (PBMCs) were isolated from buffy coats of healthy donors (German Red Cross, Dresden, Germany) using density centrifugation with Pancoll separating solution (1,077g/ml) (PanBiotech, Aidenbach, Germany). Thereafter, primary T cells were isolated using pan T cell isolation kit according to the manufacturer’s instructions (Miltenyi Biotec, Bergisch Gladbach, Germany). The detailed transduction of the genetically modified RevCAR and Dual-RevCAR T cells were described in details previously (24). During transduction, T cells were cultured with IL-15, IL-7 and IL-2 (Miltenyi Biotec). However, 20-24 h before the assays, RevCAR T cells were cultured in RPMI complete medium lacking these cytokines. The research conducted with human T cells was approved by the local ethics committee of the Medical Faculty Carl Gustav Carus, Technische Universität Dresden, Germany (EK138042014).

### Design of the RevCAR receptors

The detailed design of the RevCAR molecules was described previously (24). Briefly, the RevCAR contain three main regions; the intracellular, transmembrane and the extracellular hinge and epitope. The intracellular domain contains the CD3z signaling motif connected to CD28 costimulatory domain followed by the transmembrane region of CD28. While extracellularly, the hinge region is connected to one of the peptide epitopes, either E5B9 or E7B6 which are derived from the intracellular protein La/SS-B (25, 45). A signaling peptide of human IL-2 is located N-terminally in order to allow the transportation of the molecules to the cell surface. The Dual-RevCAR vector encodes two different RevCAR receptors: SIG RevCAR-E7B6-3z and COS RevCAR-E5B9-28. The COS RevCAR-E5B9-28 contains CD28 CSD and RevCAR-E7B6-3z contains CD3 zeta SD, respectively at their intracellular domain. The two RevCAR reading frames are separated by 2A self-cleaving peptide (P2A) and are expressed bicistronically under the control of one promoter. Both RevCARs use different hinge and transmembrane domains to avoid dimerization.

### Determining receptor density and flow cytometry analysis

The receptor density of RevCAR molecules on the surface of transduced T cells was estimated using QIFIKIT^®^ (Agilent, Santa Clara, USA) as described by the manufacturer. Similarly, the density of GD2 and EGFR on the surface of tumor cells were determined. Briefly, GD2 expression was detected using anti-GD2 mAb (clone 14G2a, BioLegend, San Diego, CA, USA), and EGFR was determined using anti-human EGFR mAb (clone AY13; BioLegend), both followed by goat-anti-mouse IgG-AlexaFlour 647™ (Thermo Fisher Scientific, Germany). As a control, mouse IgG1 isotype (clone MOPC-21; BD Biosciences, Heidelberg, Germany) or Mouse IgG2a (clone S43.10; Miltenyi Biotec) were used.

For detection of the RevTMs binding, 1x10^5^ cells were incubated with 50 ul of RevTM (40ug/ml) for 1h at 4°C. After washing with PBS, the cells were incubated with APC-conjugated anti-His Ab (clone GG11-8F3.5.1, Miltenyi Biotec). EGFR and GD2 were also detected using primary Abs mentioned above, followed by goat-anti-mouse IgG-Pacific Blue™ (Thermo Fisher Scientific). All samples were measured using a MACSQuant^®^ Analyzer and MACSQuantify^®^ software (Miltenyi Biotec).

### Expression and purification of recombinant RevTMs

The GD2-specific scFv and the EGFR nanobody domains were constructed as described previously (47, 48, 84). The sequences of these domains were first amplified by PCR with the Advantage_HF2 PCR Kit (Clontech Laboratories, Inc., CA, USA). These sequences were inserted *via* the restriction enzymes *Sfi*I/*Not*I into intermediate vector pSecTag2B, containing the sequences of the second part of the RevTM; anti-E5B9 scFv or anti-E7B6 scFv with the myc and His tags as shown in **Figure 1**. The complete RevTM sequences were cut out with *Nhe*I/*Mes*I restriction enzymes and inserted into the *Xb*aI/*HpaI* digested lentiviral vector p6NST50. The original empty vector pSecTag2B was purchased from Invitrogen GmbH, Karlsruhe, Germany. For creation of GD2-IgG4-5B9 RevTM, GD2 scFv flanked with *Sfi*I/*Mre*I restriction sites was cloned into p6NST50 vector containing the IgG4 hinge and constant domains along with sortase recognition site and 6xHis tag using the *Sfi*I and *Mre*I restriction enzymes. The complete RevTM sequences were cut out with *Nhe*I/*Mes*I restriction enzymes and inserted into the *Xba*I/*Hpa*I digested lentiviral vector p6NST50. All restriction enzymes were purchased from ThermoFischer scientific.

These vectors were used to produce lentiviral particles encoding the sequences of the RevTMs, which were then used to transduce 3T3 cells to permanently express the RevTMs. Supernatants from transduced cell lines were collected and the respective RevTM was purified using Ni-NTA affinity chromatography according to manufacturer’s instructions (Qiagen, Hilden, Germany). Purified proteins were separated using SDS-PAGE and detected using Quick Coomassie^®^ Stain (Serva, Heidelberg, Germany) or immunoblotting as described previously (66, 85).

### Cytotoxicity assay

Luminescence-based assay was used to determine the RevCAR T cell-mediated tumor cytotoxicity. For that purpose, cell lines expressing firefly luciferase were used (U251 Luc, U343 Luc and HEK 293T Luc). The cytotoxicity assays were performed at E:T ratio of 1:4 by co-culturing 1x10^4^ RevCAR T cells and 4x10^4^ tumor cells in the absence or presence of 25 nM or a range of concentrations of the RevTMs, as indicated in each experiment. On the other hand, the Dual-RevCAR T cells were used at E:T ratio of 1:2, where 2x10^4^ RevCAR T cells were co-cultured with 4x10^4^ tumor cells and 25 nM of each RevTM, or in combination (RevTM EGFR-7B6 with either RevTM GD2-IgG4-5B9 or GD2-5B9) in a total of 200 µl RPMI. After 18-20 h of incubation, the luminescence was measured and the specific lysis was calculated as described previously (84).

### Cytokine measurement

To determine cytokine secretion, tumor cells were incubated with RevCAR T cells in the absence or presence of the corresponding RevTM (25nM). After 18-20 h of incubation, supernatants were collected and analyzed using ELISA kit (BD Biosciences, Heidelberg, Germany) according to the manufacturer’s instructions. Standards ranging from 7.8 to 500 pg/mL were used in order to quantify each cytokine. All samples were appropriately diluted to ensure that their concentration falls within the standard curve.

### *In vivo* killing experiment

Animal experiments were performed in accordance with the guidelines of the German Regulations for Animal Welfare, approved by the local Ethical Committee for Animal Experiments (reference numbers DD24.1-5131/449/67 and DD24.1-5131/449/49).

*In vivo* co-injection experiment was performed in five groups of 8 weeks old female NXG-immunodeficient mice (NOD-Prkdcscid-IL2rgTm1/Rj, JANVIER LABS, Le Genest-Saint, France) with 5 mice in each group. The first group was injected with 1x10^6^ cells U251 Luc cells, the second group was injected with a mixture of 1x10^6^ cells U251 Luc cells with 1x10^6^ Dual-RevCAR T cells at 1:1 ratio. The third and fourth group were injected with the same mixture as the second group but with the addition of 150 pmol/100 ul of RevTM GD2-IgG4-5B9 or EGFR-7B6 respectively. The fifth group was co-injected with U251 Luc cells, Dual-RevCAR T cells, and a combination of RevTMs GD2-IgG4-5B9 and EGFR-7B6 using the same quantities as mentioned above. A total volume of 100 µl in PBS was administrated subcutaneously in the right thigh of the mice. Prior to optical imaging, mice were anesthetized as described before (84), and injected with 200 µL XenoLight D-Luciferin Potassium Salt (15 mg/mL) (PerkinElmer LAS GmbH, Rodgau, Germany). After ten minutes, luminescence was detected using the *In Vivo* Xtreme Imaging System (Bruker, Bremen, Germany) with exposure time of 5 min. The measurement was performed over a period of nine days.

### RevTM radiolabeling

The bispidine ligand was synthesized according to the literature (86). For functionalization of the RevTMs, an aromatic isothiocyanate group was introduced on the C9-position of the bispidine ligand forming a thiourea bond (87, 88). Copper-64 labeling gave quantitative radiochemical yields (>99%) with final molar activities of ~42 MBq/nmol for [^64^Cu]Cu-bispidine-RevTM-GD2-7B6 and ~60 MBq/nmol for [^64^Cu]Cu-bispidine-RevTM-GD2-IgG4-5B9.

### PET imaging

Small animal positron emission tomography (PET) was performed using the nanoScan PET/CT scanner (Mediso Medical Imaging Systems, Budapest, Hungary). Each animal (NXG mice, n = 2) received an intravenous injection of 10 MBq ^64^Cu-labeled RevTMs delivered in 0.2 mL of 0.154 mol/L NaCl(aq) through a tail vein catheter. Images were recorded at the following time points after RevTM injection: 0−1 h, 5 h (4.5−5 h), 24 h (23−25 h), and 48 h (47−49 h). With each PET scan, a corresponding CT image was recorded and used for anatomical referencing and attenuation correction. Binning, framing, and image reconstruction were performed as reported previously (89). Images were post-processed and analyzed using ROVER (ABX, Radeberg, Germany) and displayed as maximum intensity projections (MIPs) at indicated time points and scaling. Three-dimensional volumes of interest (VOI) were created applying fixed thresholds for delineation of tumor (20%), heart (80%), kidneys (50%), and liver (80%). Standardized uptake values (SUV) were determined and reported as SUVmean (VOI-averaged).

### Statistical analysis

Statistical analysis was performed with GraphPad Prism 9.0 (La Jolla, CA, USA). Statistical significance was determined by one-way ANOVA with Dunnett’s multiple comparison test. P-values below 0.033 were considered statistically significant.

## Data availability statement

The original contributions presented in the study are included in the article/**Supplementary Material**. Further inquiries can be directed to the corresponding author.

## Ethics statement

The animal study was reviewed and approved by the local Ethical Committee for Animal Experiments (reference numbers DD24.1-5131/449/67 and DD24.1-5131/449/49).

## Author contributions

AF, MB, NM and HS contributed to conception and formal analysis. HS, NM, CA, LL, AK, KS, MF, MB and AF contributed to methodology and investigation. AF, MB, CR, BB and JP provided critical material and resources. HS, NM, MU, MK, MT, WD-C, CN performed experiments and curated data. NM and HS wrote the original draft of the manuscript. MU, MK, MT, WD-C, CN, CA, LL, AK, KG, BB, CR, JP, MF, MB, AF reviewed and edited the original draft of the manuscript. NM, HS and MU visualized the data. AF, MB and NM supervised the project. All authors contributed to the article and approved the submitted version.
