# Supplementary material for: Specific and safe targeting of glioblastoma using switchable and logic-gated RevCAR T cells
Source: Front Immunol. 2023 Apr 14;14:1166169. doi: 10.3389/fimmu.2023.1166169 (PMC10145173; doi:10.3389/fimmu.2023.1166169)
Supplement: Supplementary file 1 [file DataSheet_1.pdf]

Supplementary Material

Specific and safe targeting of glioblastoma using switchable and logic-gated RevCAR T cells

Haidy A. Saleh, Nicola Mitwasi, Martin Ullrich, Manja Kubeil, Magali Toussaint, Winnie Deuther-Conrad, Christin Neuber, Claudia Arndt, Liliana R. Loureiro, Alexandra Kegler, Karla Elizabeth González Soto, Birgit Belter, Claudia Rössig, Jens Pietzsch, Marcus Frenz, Michael Bachmann* and Anja Feldmann.

*** Correspondence:** Michael Bachmann: m.bachmann@hzdr.de

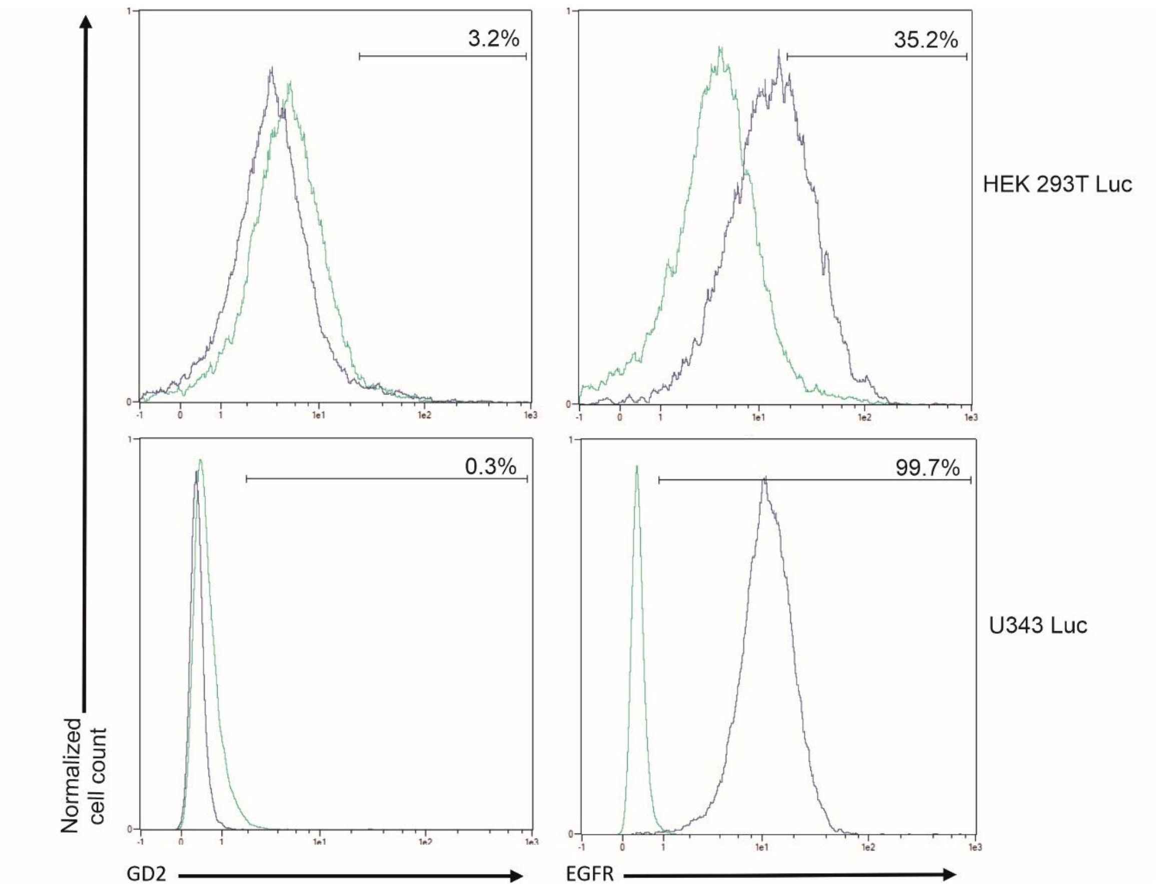

Supplementary Figure 1. EGFR and GD2 expression on glioblastoma cell lines. Expression of EGFR and GD2 on HEK 293T Luc or U343 Luc cells was detected using anti-GD2 and anti-EGFR mAb, and Alexa-Fluor 647- or Pacific Blue-conjugated goat anti-mouse secondary Ab. Flow cytometry data are represented as histograms, where light lines indicate the isotype control and dark lines indicate the stained cells.
